# Icariin protects against sodium azide—induced neurotoxicity by activating the PI3K/Akt/GSK-3β signaling pathway

**DOI:** 10.7717/peerj.8955

**Published:** 2020-04-20

**Authors:** Ying Zhang, Nanqu Huang, Hao Lu, Juan Huang, Hai Jin, Jingshan Shi, Feng Jin

**Affiliations:** 1 Key Laboratory of Basic Pharmacology and Joint International Research Laboratory of Ethnomedicine of Ministry of Education, Zunyi Medical University, Zunyi, Guizhou, China; 2 Department of Pharmacy, People’s Hospital of Zhongmu, Zhengzhou, Henan, China; 3 National Drug Clinical Trial Institution, The Third Affiliated Hospital of Zunyi Medical University (The First People’s Hospital of Zunyi), Zunyi, Guizhou, China; 4 School of Public Health, Zunyi Medical University, Zunyi, Guizhou, China; 5 Institute of Digestive Diseases of Affiliated Hospital, Zunyi Medical University, Zunyi, Guizhou, China

**Keywords:** Neurodegenerative diseases, Alzheimer’s disease, Icariin, Sodium azide, Phosphoinositide 3-kinase, Protein kinase B, Glycogen synthase kinase-3β

## Abstract

**Background:**

Icariin (ICA) is one of the major active flavonoids extracted from the traditional Chinese herb *Epimedium brevicornum* Maxim and has been shown to have neuroprotective effects. This study was designed to investigate the effect of ICA on sodium azide (NaN_3_)-induced rat adrenal pheochromocytoma (PC12) cell damage and to further examine the underlying mechanisms.

**Methods:**

To explore its possible mechanism, we used NaN_3_ (50 mM)-induced neuronal PC12 cell damage. Cell viability was evaluated by CCK-8 and lactate dehydrogenase (LDH) assays. Mitochondrial membrane potential (MMP) was detected by JC-1. Glucose concentration was assessed by the glucose oxidase method. The role of ICA in the PI3K/Akt/GSK-3β signaling pathway was explored by Western blotting.

**Results:**

The results indicate that pretreatment with ICA reduced NaN_3_-induced cell damage and significantly reduced the leakage rate of LDH in PC12 cells. ICA pretreatment increased the MMP and a decrease in glucose concentration indicate increased glucose consumption. Furthermore, the protein levels of p-PI3K (p85), PI3K-110α, p-Ser473-Akt and p-Ser9-GSK-3β were markedly decreased in PC12 cells after NaN_3_ treatment for 24 h, whereas these effects were reverted after pretreatment with ICA. Tau phosphorylation at the Ser396/404 and Thr217 sites was significantly decreased by pretreatment with ICA.

**Conclusions:**

These results suggest that ICA protects against NaN_3_-induced neurotoxicity in PC12 cells by activating the PI3K/Akt/GSK-3β signaling pathway.

## Introduction

Mitochondria act as cellular power plants that provide approximately 95% of the energy required for cellular activity (Huang & Romani, 1991). The activities of various nerve cells, such as nerve conduction, synaptic transmission, axonal transport, rely mainly on mitochondria-generated adenosine 5′-triphosphate (ATP) to provide energy (Swerdlow & Khan, 2004). Therefore, mitochondrial dysfunction plays an important role in neurodegenerative diseases (especially Alzheimer’s disease (AD)) (Eckert et al., 2014; Shoshan-Barmatz et al., 2018). Furthermore, the level of amyloid β (Aβ) aggregation, abnormal phosphorylation of Tau protein, synaptic function and neuronal apoptosis are closely related to mitochondrial dysfunction (Swerdlow, Burns & Khan, 2010). An autopsy survey showed AD patient energy metabolism disorders and mitochondrial respiratory dysfunction in brain tissue and the activity of mitochondrial respiratory chain complex IV was significantly decreased (Garcia-Escudero et al., 2013). Disturbances in the energy metabolism of the brain only affects the functional state of the brain and mitochondrial function but also leads to increased production of mitochondrial reactive oxygen species (ROS), resulting in oxidative stress (Cheignon et al., 2018; Planel et al., 2004; Wang et al., 2014; Zhu et al., 2004). Long term severe oxidative stress causes abnormalities in the phosphoinositide 3-kinase/protein kinase B/glycogen synthase kinase-3β (PI3K/Akt/GSK-3β) signaling pathway (Ali et al., 2018; Wu et al., 2020). Inactivated Akt opens the mitochondrial permeability transition pore (mPTP) by promoting GSK-3β (Arrázola et al., 2017), and the opened mPTP triggers calcium overload and the uncoupling of the respiratory chain, resulting in a reduction in ATP production and a significant increase in ROS production (Yang et al., 2017). These reactions form a vicious cycle, leading to further exacerbation of mitochondrial dysfunction. It is interesting to note that GSK-3β is also the most important protein kinase that catalyzes the phosphorylation of Tau (Zhang et al., 2018). Therefore, the above process will also lead to Tau protein hyperphosphorylation, forming a neurofibrillary tangle, which causes a cellular process that kills neurons (Hallinan et al., 2019). Therefore, the strategy of protecting mitochondria by activating the PI3K/Akt/GSK-3β signaling pathway is an effective method for the treatment of neurodegenerative diseases.

Icariin (ICA) is extracted from the traditional Chinese herb *Epimedium brevicornum* Maxim and has been used to improve cognitive impairments through different mechanisms in diverse animal and cell models of AD, which is a neurodegenerative disease (Klingelhoefer & Reichmann, 2015; Mo et al., 2016; Xiong et al., 2016). Relevant research results have shown that ICA significantly improves mitochondrial structure and function in a triple-transgenic mouse model of AD (Chen et al., 2016). Therefore, we hypothesize that ICA improves disordered brain mitochondrial energy metabolism, and its mechanism may be related to the PI3K/Akt/GSK-3β signaling pathway.

Therefore, to verify our hypothesis, a mitochondrial damage model in PC12 cells (the PC12 cells used in this study are neuron-like cells that were derived from a transplantable adrenal pheochromocytoma, a commonly used nerve cell line (Huang et al., 2019)) induced by the mitochondrial complex IV inhibitor sodium azide (NaN_3_) (Chen et al., 2013; Ishiguro et al., 2001; Szabados et al., 2004) was used to evaluate the protective effect of ICA against NaN_3_-induced mitochondrial damage and its possible mechanisms were explored.

## Materials and Methods

### Reagents

Reagent grade ICA (purity ≥ 98% by HPLC analysis) was obtained from Nanjing Zelang Medical Technology Corporation Ltd. (Nanjing, China) and dissolved in dimethyl sulfoxide (DMSO); the final concentration of DMSO in the media was less than 0.1% (v/v). NaN_3_ (A0639) was purchased from Amresco (Solon, OH, USA). RIPA buffer (high) (R0010) and protein phosphatase inhibitor (P1260) were purchased from Solarbio Life Science (Beijing, China). A glucose oxidase assay kit (E10160) and antibodies against GSK-3β (9315), p-Ser9-GSK-3β (9323) and p-PI3K (p85) (4228) were obtained from Cell Signaling Technology (Beverly, MA, USA). Goat anti-mouse IgG-HRP (SA00001-1), goat anti-rabbit IgG-HRP (SA00001-2), and antibodies against GAPDH (60004-1-Ig), and PI3K p110α (21890-1-AP) were obtained from Proteintech Group (Wuhan, China). Antibodies against PHF1 (ab184951) and PI3K (ab86714) were obtained from Abcam (Cambridge, MA, USA). PageRuler prestained protein ladder (26616) and antibodies against p-T217 (44-744) and TAU-5 (MA5-12808) were obtained from Thermo Fisher Scientific (Waltham, MA, USA). RPMI 1640 HyClone™ cell culture medium (SH30809.01) was purchased from GE Healthcare (Chicago, IL, USA).

### Cell culture and treatment

Rat adrenal pheochromocytoma PC12 cells were purchased from the American Type Culture Collection (Rockville, MD, USA).

The cells were cultured in RPMI 1640 medium supplemented with 10% horse serum (16050-122; Gibco™, Carlsbad, CA, USA), 5% FBS (16000-044; Gibco™, Carlsbad, CA, USA), penicillin (100 U/ml) and streptomycin (100 μg/ml) (P1400; Solarbio™, Beijing, China) and maintained at 37 °C and 5% CO_2_. The PC12 cells (1.5 × 10^5^ cells/mL) were plated overnight at 37 °C for 24 h. The cells were pretreated with ICA for 2 h and thereafter exposed to 50 mM NaN_3_ (dissolved in saline) for an additional 24 h. Then, the cells were subjected to subsequent experiments and assays.

### Cell viability determination

Cell viability was detected by CCK-8 assay (CA1210; Solarbio™, Beijing, China); which uses (2-(2-methoxy-4-nitrophenyl)-3-(4-nitrophenyl)-5-(2,4-disulfophenyl)-2H-tetrazolium, monosodium salt), to produces a water-soluble formazan dye upon bioreduction in the presence of an electron carrier. Briefly, PC12 cells (1.5 × 10^5^ cells/mL) were seeded in each well of a 96-well plate for 24 h. After the end of the treatments, CCK-8 solution (10 μL) was added to each well of the 96-well plate and incubated for 2 h at 37 °C. The absorbance was measured at 450 nm with an automatic microplate reader (Multiskan™ GO, Waltham, MA, USA).

### Measurement of lactate dehydrogenase release

The effects of ICA on the LDH leakage rate in NaN_3_-induced PC12 cells were detected by an LDH (C0016; Beyotime™, Beijing, China) assay kit. Briefly, according to the manufacturer’s instructions, after treatment, the supernatant of each well of a 96-well plate was collected. The positive control showing total release (100% LDH release) was treatment of cells with an LDH release agent (C0016-1). The optical density was measured at 490 nm with an automatic microplate reader (Multiskan™ GO, Waltham, MA, USA).

### Detection of mitochondrial membrane potential

MMP was measured by using a JC-1 (a fluorescent cationic probe) kit (C2006; Beyotime™, Beijing, China). Briefly, PC12 cells (1.5 × 10^5^ cells/mL) were seeded in 24-well plates, and after treatment, the cells were washed with PBS three times. A total of 0.5 ml culture medium and 0.5 ml JC-1 staining solution were added and incubated at 37 °C for 20 min. Carbonyl cyanide 3-chlorophenylhydrazone (CCCP) was used as the control of MMP decrease (Dong et al., 2016). The cells were rinsed with binding buffer twice and observed under an inverted fluorescence microscope (IX73; Olympus™, Shinjuku City, Tokyo, Japan).

### Glucose consumption rate

The concentration of glucose was detected using the glucose oxidase method (Li et al., 2015). The assay kit (E1010; Applygen Technologies, Beijing, China) was purchased from Applygen Technologies (Beijing, China). After treatment, the PC12 cells were collected, and the concentration of total protein was extracted using a total protein extraction kit (P1250; Applygen, Beijing, China) and detected using a BCA protein assay kit (P0012; Beyotime, Beijing, China). Then, according to the instructions, the absorbance was measured at 570 nm with an automatic microplate reader and calculated against a glucose standard curve.

### Western blot assay

After 24 h of treatment with different concentrations of ICA or 50 µM NaN_3_ at 37 °C, the cells were harvested and lysed with RIPA lysis buffer, followed by centrifugation at 14,000 rpm at 4 °C for 10 min. The total protein concentration was measured by BCA protein assay kit (P0012; Beyotime, Beijing, China). Then, the proteins were heat-denatured at 100 °C for 5 min. Equal amounts of total protein (15 µg per lane) were loaded. And we used 8–12% SDS-PAGE to separate the proteins. The gel was run at 60 V for the stacking gel and 100 V to separate the proteins until the dye ran off the bottom of the gel. Then, the sandwich was transferred to polyvinylidene difluoride (PVDF) membranes for western blot procedure at 4 °C in 1× buffer at 220 mA for 90 min. Following three washes with TBST. Then, 5% nonfat milk was used for blocking. Then, the cells were incubated with the following primary antibodies at 4 °C for 12 h: anti-PI3K-110α (1:1,000), anti-p-PI3K (p85) (1:1,000), anti-PI3K (1:1,000), anti-Akt (1:1,000), anti-p-Ser473-Akt (1:1,000) anti-p-GSK-3β-Ser9 (1:1,000), anti-GSK-3β (1:1,000), anti-PHF-1 (1:5,000), anti-p-T217 (1:1,000) and anti-TAU-5 (1:200). Following three washes with TBST, the membranes were incubated with secondary antibody goat anti-rabbit IgG-HRP (1:2,000) at room temperature for 1 h. Membranes were developed using hydrogen peroxide and Supersignal West Pico Luminol (Pierce; Thermo Fisher Scientific, Waltham, MA, USA). Finally, the membranes were visualized using chemiluminescence reagent ECL Plus (E003-100; 7Sea Biotech, Shanghai, China).

### Statistical analysis

All data are expressed as the mean ± SEM. The data were analyzed statistically by SPSS 22.0 statistics software via one-way ANOVA. When ANOVA test results for all data were significant, post hoc least significant difference (LSD) tests were used to determine individual differences. Statistical significance was set as *P* < 0.05.

## Results

### ICA attenuated NaN_3_-induced damage in PC12 cells

**Figure 1:**
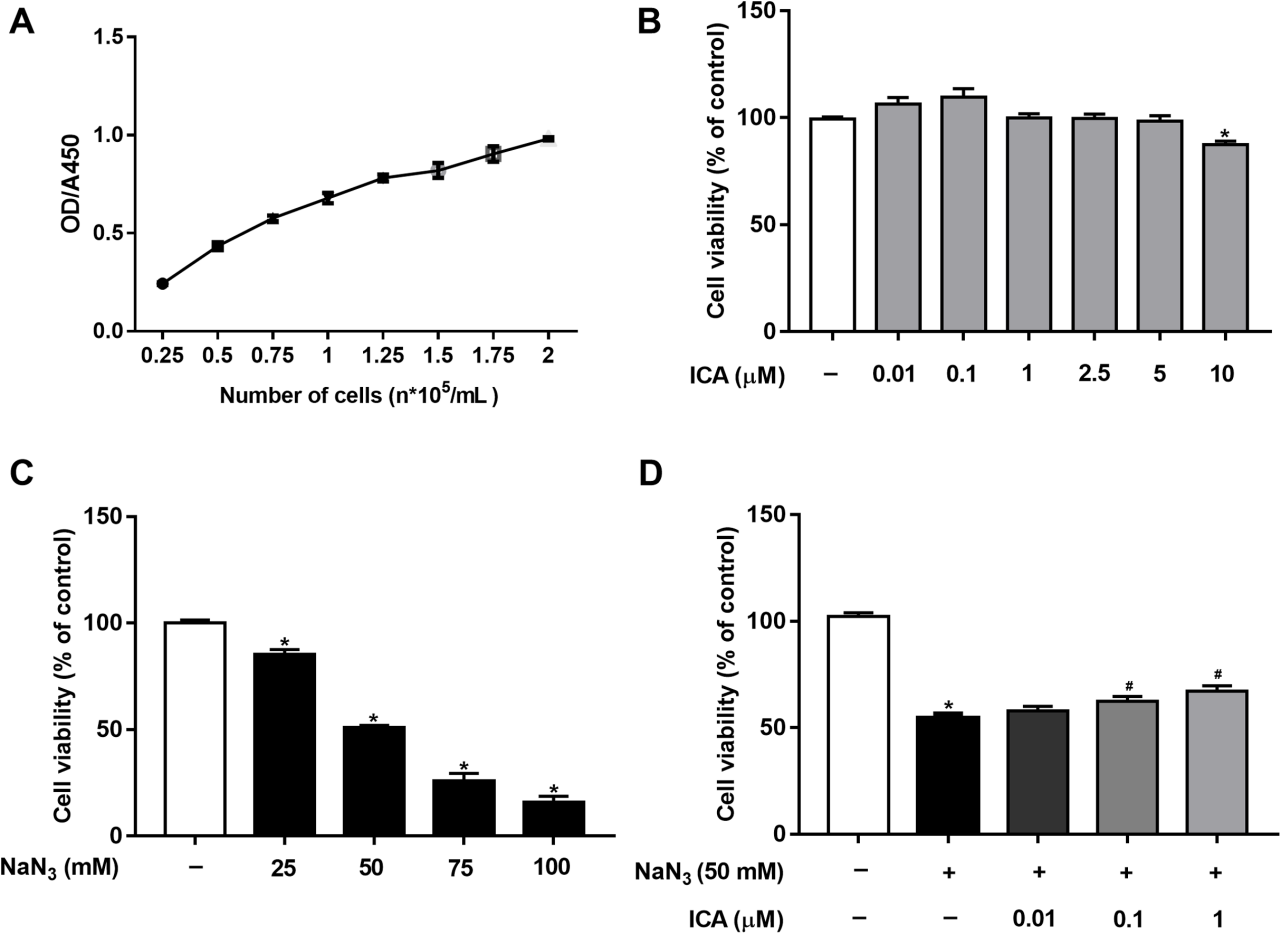
Protective effect of ICA on NaN_3_-injured PC12 cells. (A) PC12 cell growth curve. (B) Effects of simple ICA pretreatment on PC12 cells (*P < 0.05 vs. control). (C) Effects of different concentrations of NaN_3_ on PC12 cell activity (*P < 0.05 vs. control). (D) Effects of ICA on NaN_3_-inhibited growth of PC12. The data are shown as the mean ± SEM, *n* = 4 (*P < 0.05 vs. the control group, ^#^*P* < 0.05 vs. the NaN_3_ group).

Cell viability was detected by CCK-8 assay. First, based on our previous study (Huang et al., 2019) and OD/A450 of approximately 0.8, the appropriate cell seeding concentration was 1.5 × 10^5^ cells/mL (Fig. 1A). Then, we determined that ICA (0.01–5 μM) had no significant toxic effects on PC12 cells, except for 10 μM ICA for 24 h (Fig. 1B). Combined with previous studies by our laboratory, ICA at concentrations of 0.01–1 μM was used in the subsequent experiments. As shown in Fig. 1C, NaN_3_ decreased cell viability, and the viability of PC12 cells decreased by approximately 50% compared with that of the control at a final concentration of 50 mM. Furthermore, we investigated whether ICA protected PC12 cells from NaN_3_-induced reductions in cell viability. The WST-8 contained in the CCK-8 reagent is reduced to a highly water-soluble yellow formazan product by dehydrogenase found in the mitochondria of living but not dead cells. Therefore, cell viability can be indirectly determined by detecting the absorbance of the lysed formazan. The results indicated that pretreatment with ICA reduced NaN_3_-induced cell damage (Fig. 1D).

### Effect of ICA on NaN_3_-induced LDH release in PC12 cells

**Figure 2:**
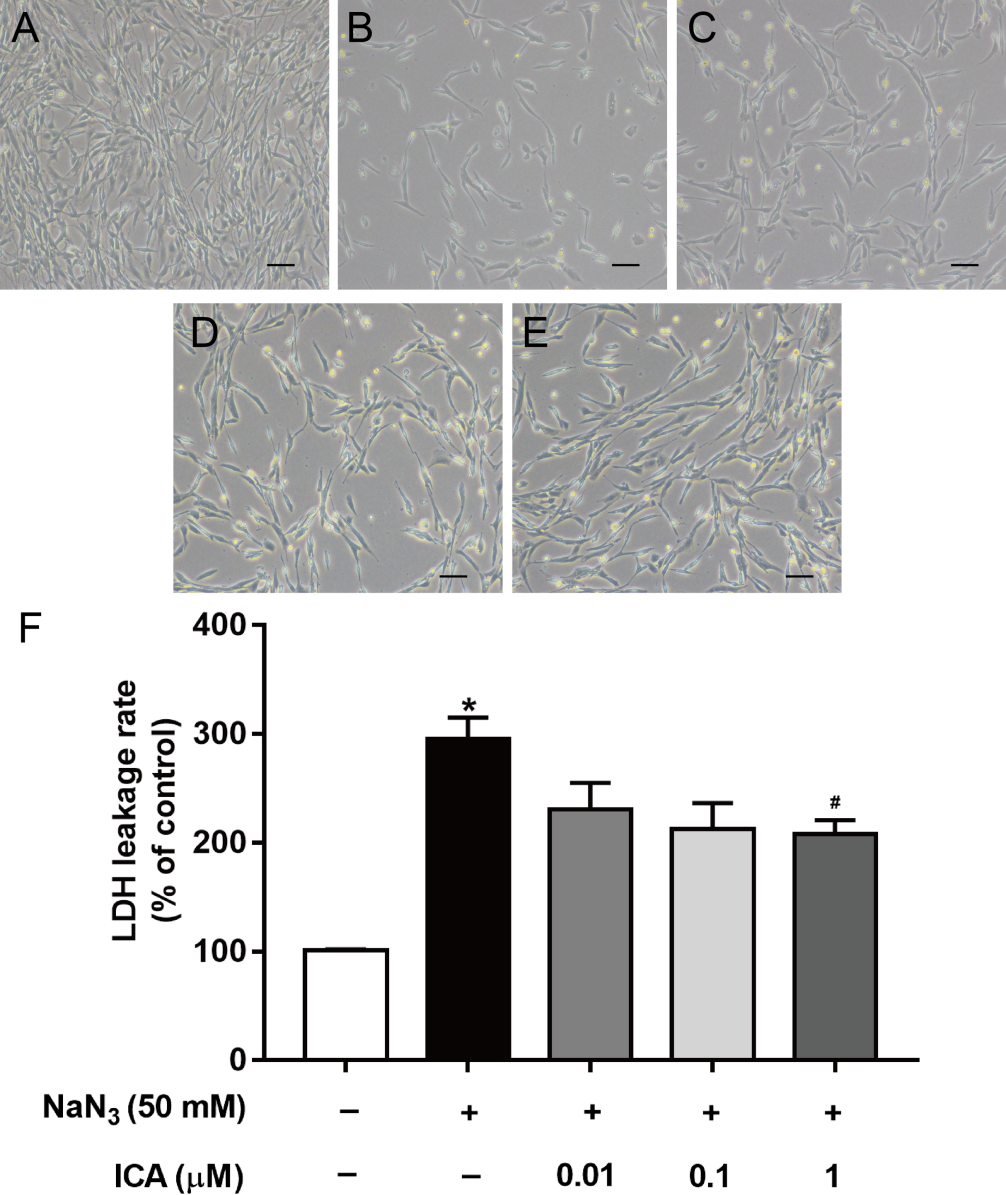
Effects of ICA on the LDH leakage rate of NaN_3_-injured PC12 cells. (A) Control group. (B) Model group (NaN_3_ 50 mM). (C) ICA-L group (NaN_3_ 50 mM + ICA 0.01 μm). (D) ICA-M group (NaN_3_ 50 mM + ICA 0.1 μm). (E) ICA-H group (NaN_3_ 50 mM + ICA 1 μm) (representative images of PC12 cells, scale bar is 20 μm). (F) Effects of ICA on the LDH leakage rate of NaN_3_-injured PC12 cells. The data are shown as the mean ± SEM, *n* = 4 (*P < 0.05 vs. the control group, ^#^*P* < 0.05 vs. the NaN_3_ group).

We found that ICA pretreatment prevented NaN_3_-induced morphological damage (Figs. 2A–2E). LDH leakage is an indicator of cell membrane integrity. When the cell membrane is damaged, intracellular LDH is released into the cell culture medium. Therefore, the leakage rate of LDH reflects the level of cell damage. The results showed that LDH release was higher in NaN_3_-treated cells than in control cells, while ICA significantly reduced the LDH leakage rate in PC12 cells (Fig. 2F).

### Effect of ICA on the NaN_3_-induced change in MMP in PC12 cells

**Figure 3:**
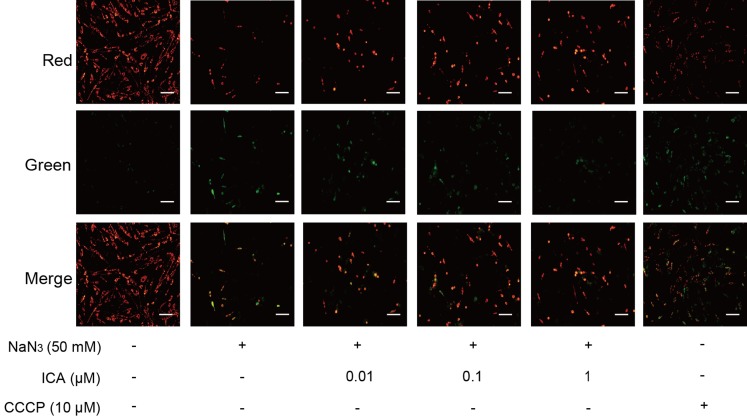
Effect of ICA on MMP in NaN_3_-injured PC12 cells. Representative images of JC-1 staining in PC12 cells in the different groups. Red fluorescence represents increased MMP, green fluorescence represents reduced MMP and CCCP is a mitochondrial electron transport chain inhibitor that serves as a positive control for MMP depolarization (scale bar is 20 μm, *n* = 3).

The change in MMP was assessed by using JC-1. The change in MMP is detected by the change from red fluorescence to green fluorescence. As shown in Fig. 3, NaN_3_-treated cells showed a decrease in MMP in comparison with that of the control, which was consistent with the CCCP group. ICA pretreatment increased red fluorescence and decreased green fluorescence. These results suggest that ICA pretreatment increases the MMP of PC12 cells.

### ICA ameliorated the NaN_3_-induced reduction in the glucose consumption rate in PC12 cells

**Figure 4:**
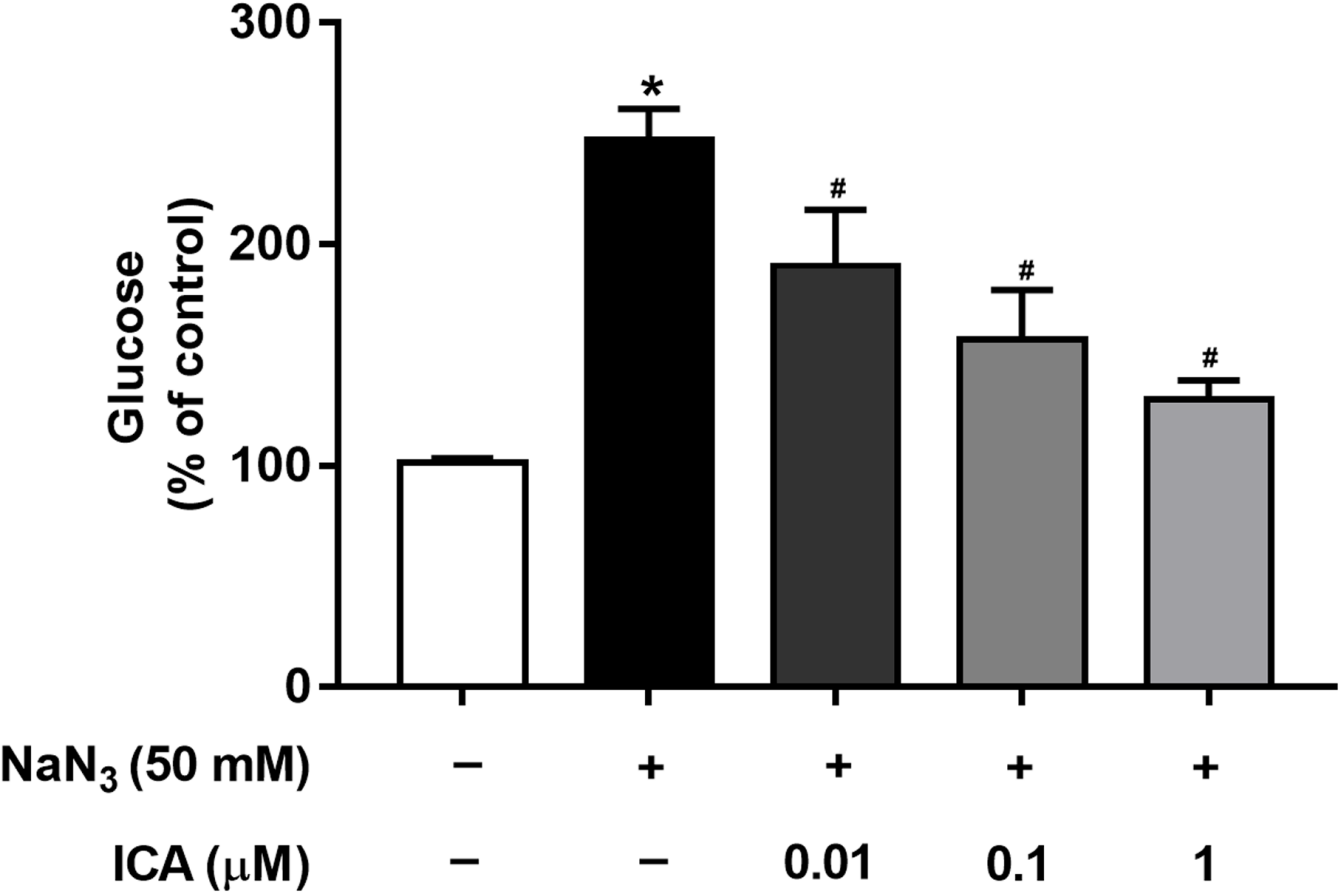
Effects of ICA on glucose levels in NaN_3_-injured PC12 cells. The data are shown as the mean ± SEM, *n* = 4 (*P < 0.05 vs. the control group, ^#^*P* < 0.05 vs. the NaN_3_ group).

Mitochondria are cellular power plants and the energy required for the activity of neurons is mainly provided by the glucose metabolism of brain mitochondria. To investigate the glucose metabolism level, we examined the glucose content in PC12 cells. The NaN_3_-administered group exhibited a reduction in glucose consumption compared with that of the control group, leading to an increase in glucose concentration. However, ICA pretreatment ameliorated this effect (Fig. 4).

### ICA activated the PI3K/Akt/GSK-3β signaling pathway in PC12 cells

**Figure 5:**
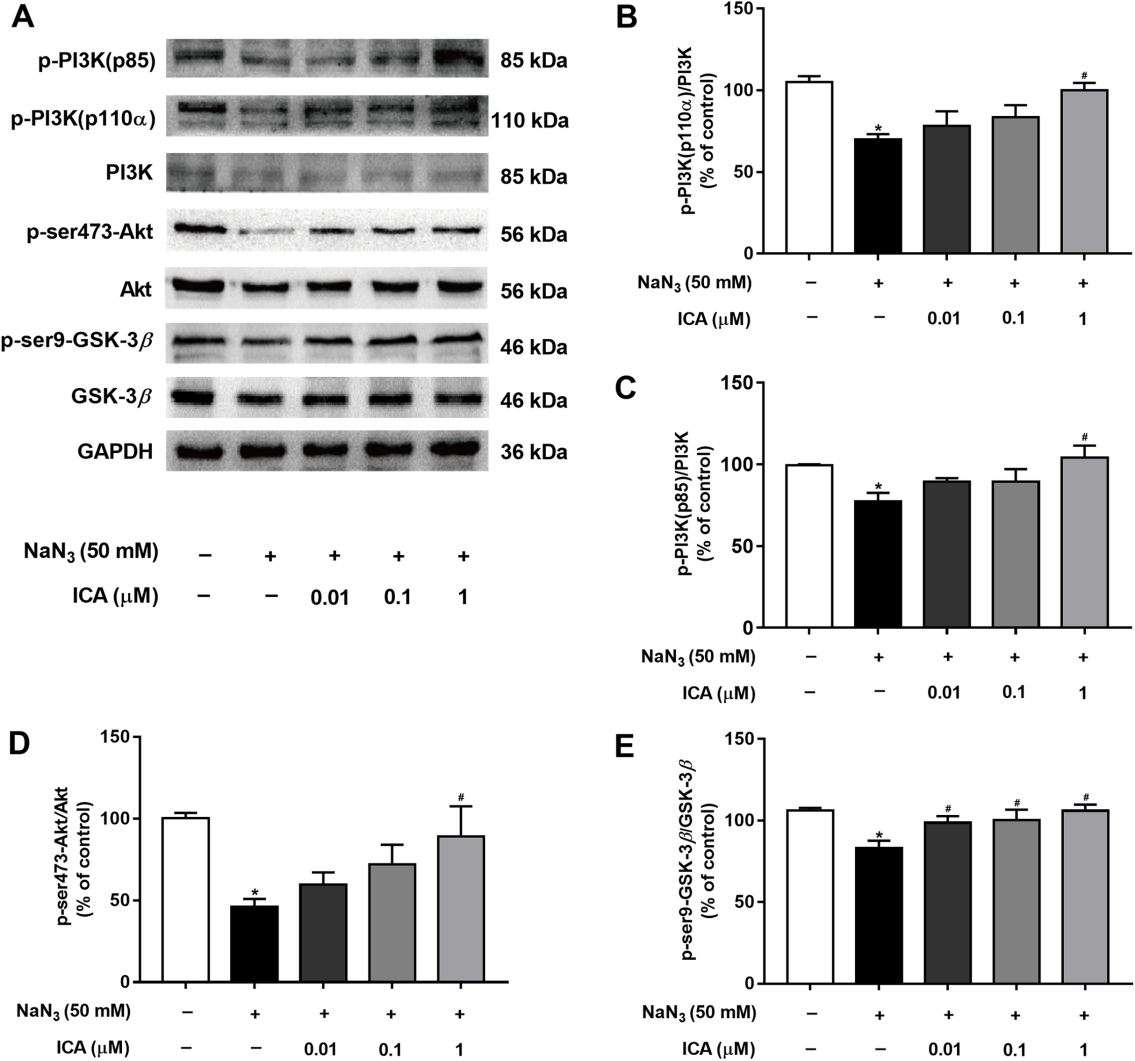
Effect of ICA on the PI3K/Akt/GSK-3β signaling pathway in NaN_3_-injured PC12 cells. (A) Representative bands showing PI3K-110 α, p-PI3K (p85), PI3K, p-ser473-Akt, Akt, p-ser9-GSK-3β and GSK-3β in PC12 cells in the different groups. (B) Quantitative analysis of PI3K-110α levels. (C) Quantitative analysis of p-PI3K (p85) levels. (D) Quantitative analysis of p-Ser473-Akt levels. (E) Quantitative analysis of p-ser9-GSK-3β levels. The data are shown as the mean ± SEM, *n* = 45 (*P < 0.05 vs. the control group, ^#^*P* < 0.05 vs. the NaN_3_ group).

To determine whether the PI3K/Akt/GSK-3β signaling pathway is involved in the protective effects of ICA against NaN_3_-induced neuronal damage, we examined the expression of related proteins. As shown in Fig. 5, the protein levels of p-PI3K (p85), PI3K-110α, p-Ser473-Akt and p-Ser9-GSK-3β were markedly decreased in PC12 cells after NaN_3_ treatment for 24 h, whereas these effects were reverted after pretreatment with ICA. These results suggest that the protective effect of ICA on NaN_3_-induced mitochondrial dysfunction is related to the PI3K/Akt/GSK-3β signaling pathway (Fig. 5).

### ICA reduced the levels of tau phosphorylation

**Figure 6:**
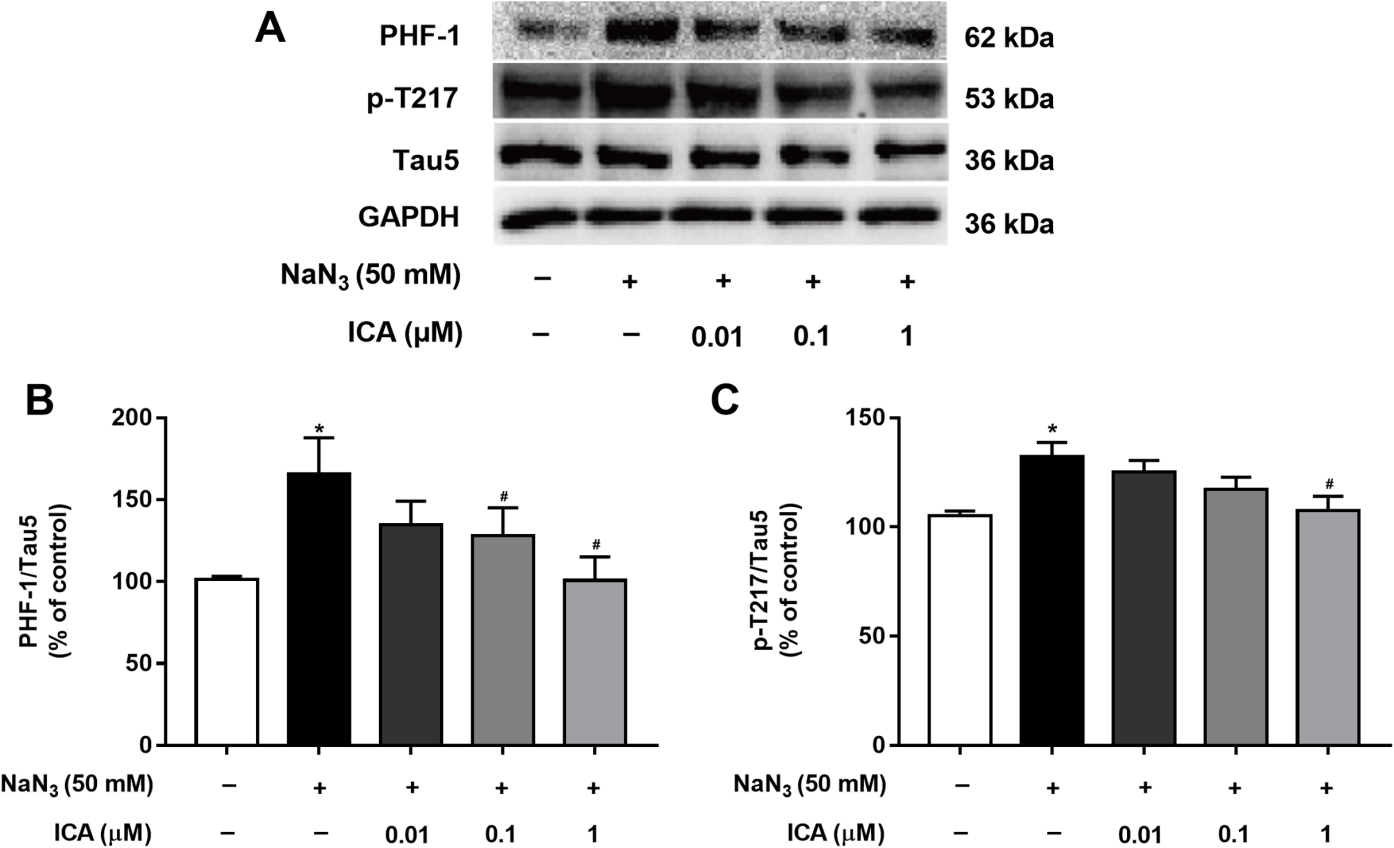
Effect of ICA on the phosphorylation level of Tau in NaN_3_-injured PC12 cells. (A) Representative bands showing PHF-1 and p-T217 in PC12 cells in the different groups. (B) Quantitative analysis of PHF-1 levels. (C) Quantitative analysis of p-T217 levels. ****Phosphorylated Tau was normalized to total Tau. The data are shown as the mean ± SEM, *n* = 5 (*P < 0.05 vs. the control group, ^#^*P* < 0.05 vs. the NaN_3_ group).

Excessive activation of GSK-3β promotes abnormal hyperphosphorylation of Tau. Therefore, we detected the phosphorylation level of Tau in PC12 cells at PHF-1 (identified Ser396/404 sites) and p-T217. As shown in Fig. 6, Tau phosphorylation at the Ser396/404 and Thr217 sites was significantly higher in NaN_3_-treated cells than in control cells. In contrast, the phosphorylation of Tau at Ser396/404 and Thr217 was decreased by pretreatment with ICA.

## Discussion

NaN_3_ is a mitochondrial complex IV inhibitor and is often used as a tool for the preparation of mitochondrial damage models to study AD prevention (Chen et al., 2013; Szabados et al., 2004). Therefore, in this study, we first examined PC12 cell damage induced by NaN_3_ treatment at 25–100 mM. We chose 50 mM NaN_3_ for subsequent experiments (the cell viability inhibition rate was close to 50%). When the cell membrane is damaged, intracellular LDH is released into the cell culture medium. Therefore, the leakage rate of LDH reflects the level of cell damage. LDH assays showed that ICA protects against NaN_3_-induced neuronal PC12 cell injury. The formation of normal MMP is necessary to maintain mitochondrial function. In response to damage induced by various stimulating factors, the decrease in MMP is a specific early event in the mitochondrial apoptosis pathway. To this end, MMP levels were determined in this study. ICA pretreatment showed increased red fluorescence and decreased green fluorescence, suggesting that ICA pretreatment increased the MMP of PC12 cells. The results from the above show that ICA protects neurons against damage from NaN_3_-induced cytotoxicity. Mitochondria are key organelles for cellular energy production and are responsible for major glucose metabolism. Mitochondrial dysfunction is mainly caused by disordered mitochondrial energy metabolism due to glucose metabolism disorder (Abolhassani et al., 2017; Almeida et al., 2002). Our results demonstrate that NaN_3_ induced a reduction in glucose consumption, leading to an increase in glucose concentration. However, ICA pretreatment ameliorated this effect, further confirming that ICA protects against NaN_3_-induced PC12 cell damage through inhibition of mitochondrial dysfunction caused by glucose metabolism disorder.

The PI3K/Akt/GSK-3β signaling pathway plays an important role in the development, survival and function of neurons (Li et al., 2016; Zhang et al., 2016), which is critical for the regulation of mitochondrial function. We hypothesized that ICA improves brain mitochondrial energy metabolism disorder, and its mechanism is related to the PI3K/Akt/GSK-3β signaling pathway. Therefore, in this study, we detected the expression of proteins related to this pathway by Western blotting. The results demonstrated that exposure of PC12 cells to NaN_3_ downregulated PI3K, Akt and GSK-3β protein levels and activated PI3K-110α, p-PI3K (p85), p-Ser473-Akt and p-Ser9-GSK-3β site-specific phosphorylation in cells that were treated with ICA. The key to activating PI3K/Akt/GSK-3β to improve AD may be Tau protein (Hernandez, Lucas & Avila, 2013). Because GSK-3β and Tau are closely related, we can improve the abnormal hyperphosphorylation of Tau by regulating GSK-3β. Our findings confirmed that the phosphorylation level of Tau in NaN_3_-induced PC12 cells at PHF-1 and p-T217 was increased. ICA treatment decreased the phosphorylation level of Tau. These data suggest that ICA protects PC12 cells from NaN_3_-induced neuronal damage by activating the PI3K/Akt/GSK-3β signaling pathway to reduce Tau. Other studies have confirmed similar results: in vitro, asiatic acid (extracted from *Centella asiatica*) protects PC12 cells against Tau protein hyperphosphorylation by activating the PI3K/Akt/GSK-3β signaling pathway (Cheng et al., 2018). In vivo, intragastric administration of cornel iridoid glycoside (extracted from *Cornus officinalis*) inhibited Tau hyperphosphorylation in rats (intraventricular injection of wortmannin and GF-109203X) by inhibiting GSK-3β activity through promoting PI3K/Akt (Yang et al., 2018). Osthole (extracted from *Cnidium monnieri* (L.) Cusson) improves learning and memory function in mice by activating the PI3K/Akt/GSK-3β signaling pathway to reduce Tau (Yao et al., 2019).

## Conclusions

These results suggest that ICA protects against NaN_3_-induced neurotoxicity in PC12 cells by activating the PI3K/Akt/GSK-3β signaling pathway.

## Supplemental Information

10.7717/peerj.8955/supp-1 Supplemental Information 1 Raw data.

10.7717/peerj.8955/supp-2 Supplemental Information 2 Full-length uncropped blots.

## Additional Information and Declarations

### Data Availability

The following information was supplied regarding data availability: The raw measurements (including full-length uncropped blots) are available in the Supplemental Files.
