# Supplementary figures and images for: Icariin protects against sodium azide—induced neurotoxicity by activating the PI3K/Akt/GSK-3β signaling pathway
Source: PeerJ. 2020 Apr 20;8:e8955. doi: 10.7717/peerj.8955 (PMC7179568; doi:10.7717/peerj.8955)

p-p85


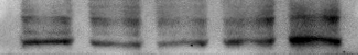


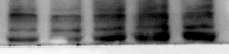


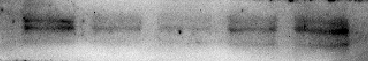

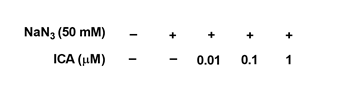


P110


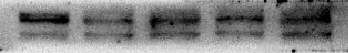


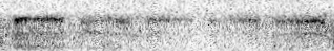


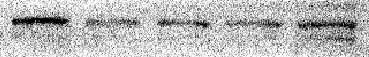


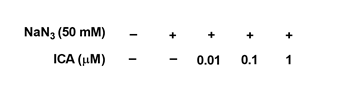


P85


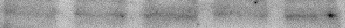


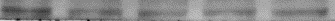


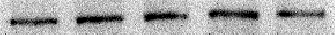


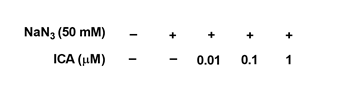


PAKT


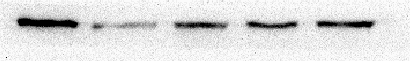


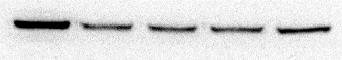


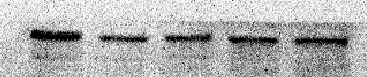


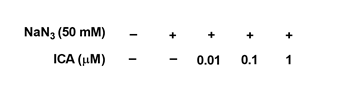


Akt


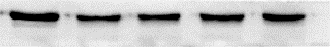


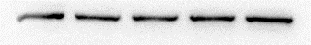


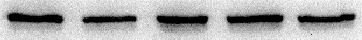


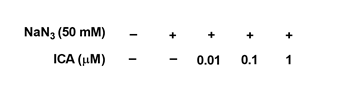


p-GSK3


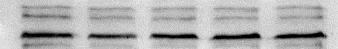


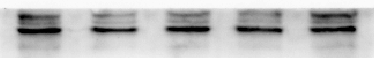


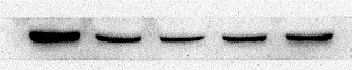


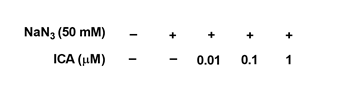


GSK3


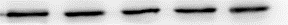


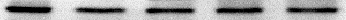


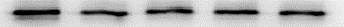


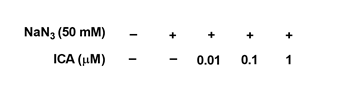


PHF


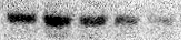


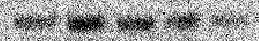


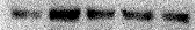


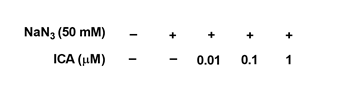


p-T217


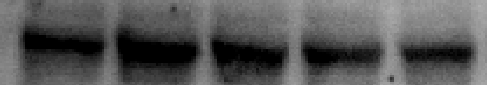


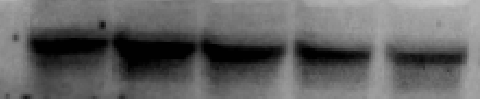


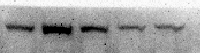


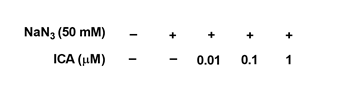


Tau


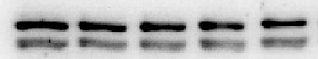


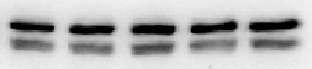


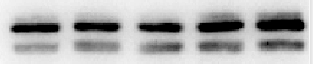


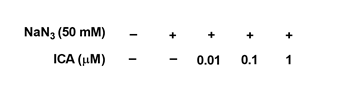


GAPDH


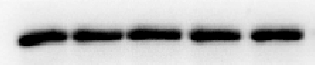


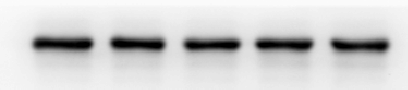


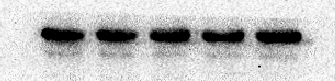


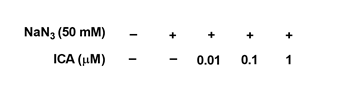

Supplement: Supplemental Information 2 [file peerj-08-8955-s002.docx]
